# Assessment of liver and spleen stiffness and hepatic steatosis by transient elastography (Fibroscan®) in type 1 Gaucher disease: a single center case–control cohort study

**DOI:** 10.1186/s13023-025-03847-5

**Published:** 2026-01-08

**Authors:** Ummu Mutlu, Bilger Cavus, Hulya Hacisahinogullari, Gulsah Yenidunya Yalin, Ozlem Soyluk Selcukbiricik, Nurdan Gul, Ayse Kubat Uzum, Kadir Demir, Refik Tanakol

**Affiliations:** 1 Istanbul Faculty of Medicine, Department of Internal Medicine, Division of Endocrinology and Metabolism, Istanbul University, Turgut Ozal Street, Capa, Sehremini Fatih Istanbul, Türkiye; 2 Istanbul Faculty of Medicine, Department of Internal Medicine, Division of Gastroenterohepatology, Istanbul University, Istanbul, Türkiye

## Abstract

**Introduction:**

Gaucher disease (GD) is a lysosomal storage disorder characterized by glucosylceramide accumulation, which may lead to liver fibrosis and cirrhosis. Enzyme replacement therapy (ERT) could reverse fibrosis. This study aimed to assess liver and spleen stiffness and hepatic steatosis in adult type 1 GD patients receiving ERT, using transient elastography (TE) (Fibroscan**®**).

**Method:**

Twenty-five type 1 GD patients were evaluated pre- and post-ERT. TE findings of GD patients on ERT were compared cross-sectionally with a control group. Liver fibrosis was defined as ≥7 kPa, and significant steatosis was defined as a Controlled Attenuation Parameter (CAP) measurement ≥ 250 dB/min. Associations between TE findings and clinical, metabolic, genetic characteristics, FIB4 (fibrosis 4) and APRI (AST to platelet ratio index) scores, were investigated.

**Results:**

Fifty-six percent of GD patients were female, with a median disease duration of 13 years. Post-ERT, body weight (57.3 vs. 63.6 kg, *p* < 0.001), body mass index (22 vs. 23.8 kg/m^2^, *p* < 0.001), and metabolic syndrome (MetS) prevalence (12% vs. 40%, *p* = 0.016) were increased. Hepatic steatosis was more frequent (32% vs. 16%). Liver fibrosis was present in 44% of GD patients, but in none of the controls. GD patients exhibited significantly higher liver (6.6 vs. 3.7 kPa; *p* < 0.001) and spleen stiffness (17.6 vs. 11.1; *p* = 0.032). Liver fibrosis was positively correlated with ALT, GGT, ferritin levels, disease duration, and delayed initiation of ERT.

**Conclusion:**

Although ERT improved fibrosis-related parameters, GD patients demonstrated higher liver and spleen stiffness. Elevated ferritin levels, longer disease duration, and delayed initiation of ERT were associated with liver fibrosis. Additionally, increased metabolic syndrome prevalence post-ERT may contribute to the development of hepatic steatosis in this patient population.

## Introduction

Gaucher disease (GD) is an autosomal recessive lysosomal storage disorder caused by pathogenic variants in the glucocerebrosidase 1 (*GBA1*) gene, located on chromosome 1q21. These mutations result in glucocerebrosidase enzyme deficiency, leading to the accumulation of glucocerebroside and other glycolipids within the lysosomes of macrophages [1, 2].

The most common form is type 1 (non-neuronopathic) GD, which is characterized by visceral and skeletal involvement, including hepatosplenomegaly, pancytopenia, and bone pain. Liver involvement presents with hepatomegaly due to glycosphingolipid accumulation. Hepatomegaly is common in GD patients and may cause abdominal pain and distension. Over time, hepatic involvement may progress to fibrosis, potentially leading to cirrhosis and portal hypertension [3–5]. Although rare, cases of hepatocellular carcinoma have also been reported in patients with GD [6, 7]. These complications have been attributed to the presence of progressive liver fibrosis. Consequently, noninvasive assessment of hepatic fibrosis represents an important step in the risk stratification and longitudinal follow-up of patients with GD.

Transient elastography (TE) by FibroScan**®** is an ultrasound-based noninvasive method for the assessment of liver and spleen stiffness and hepatic steatosis through controlled attenuation parameter (CAP) measurements. Current guidelines recommend TE as a reliable method for evaluating the severity of chronic liver diseases [8].

Glucose and lipid metabolism abnormalities, often accompanied by chronic inflammation, are common in GD. Despite clinical and metabolic improvement with enzyme replacement therapy (ERT), resolution of inflammation may lead to weight gain, potentially increasing the risk of developing metabolic syndrome (MetS) [9]. MetS, in turn, is a well-established contributor to hepatic steatosis and metabolic-associated fatty liver disease (MAFLD).

To date, only a limited number of studies have evaluated liver stiffness, spleen stiffness, and CAP measurements specifically in patients with type 1 GD [10–16]. In the present study, we retrospectively evaluated the changes in metabolic and laboratory parameters pre-ERT and post-ERT in Type 1 GD patients. Additionally, we conducted a cross-sectional comparison between GD patients and a matched control group with similar metabolic profiles, focusing on liver and spleen stiffness and hepatic steatosis.

## Material and methods

Twenty-seven adult patients diagnosed with type 1 GD and who were receiving ERT at the Department of Endocrinology and Metabolism, Istanbul Faculty of Medicine, Istanbul University, were initially deemed eligible for inclusion in the study. GD diagnosis was confirmed based on reduced glucocerebrosidase enzyme activity and the presence of pathogenic variants in the GBA1 gene. Two patients could not be contacted, and a total of 25 patients who provided informed consent were enrolled.

A control group of 25 healthy individuals whose gender characteristics, weight, and body-mass index were similar to the patient group, was also included.

Written informed consent was obtained from all of the participants, and the study protocol was approved by the Clinical Ethics Committee of Istanbul University Hospital (Approval date and number: 03.12.2024–3047128).

### Transient elastography

Transient elastography (TE) measurements were performed in all participants using the FibroScan® device (Echosens, Paris, France) by the same experienced operator (B.C.) to ensure consistency.

Two different probes were used based on body mass index (BMI): the M (medium) probe for individuals with BMI < 25 kg/m^2^, and the XL (X-large) probe for those with BMI ≥ 25 kg/m^2^. Three parameters were simultaneously assessed: controlled attenuation parameter (CAP), liver stiffness (LS), and spleen stiffness (SS). LS and SS values were expressed as the median of 10 consecutive valid measurements, reported in kilopascals (kPa). A measurement was considered valid when the interquartile range-to-median ratio was less than 30%.

Liver stiffness was assessed with the patient in the supine right lateral decubitus position, with the right arm extended above the head to maximize intercostal space. Spleen stiffness was measured with the patient in the left lateral decubitus position, similarly positioning the left arm overhead. The ultrasound probe was used to localize the spleen and exclude any space-occupying lesions prior to measurement [17, 18].

CAP evaluates steatosis quantitatively based on the degree of attenuation of ultrasound beams in the liver, and it is defined as dB/min and ranges between 100–400 dB/min [8, 19]. The CAP software is built into the transient elastography device (FibroScan, Echo-sens, France) as an add-on, and it also provides the possibility to simultaneously measure liver stiffness [19]. CAP measurement is a noninvasive, easy-to-apply method with high sensitivity and specificity used in the diagnosis of hepatic steatosis.

Elevated CAP was defined as $\geq$ 250 dB/min, and elevated LS was defined as $\geq$ 7 kPa [20]. There is no clear cut-off value for spleen stiffness. Our study aimed to establish a threshold value by comparing splenic stiffness in patients with GD and controls. However, splenic stiffness measurements could not be performed in three splenectomized patients and one patient with a history of stent implantation for splenic artery aneurysm.

### Anthropometric, demographic, metabolic, and biochemical data

Pre-ERT measurements of all GD patients were obtained from the medical records. Weight, height, BMI, waist circumference, and blood pressure were measured at the time of evaluation. Laboratory results, including fasting blood glucose, hemogram, liver enzymes, C-reactive protein, ferritin, erythrocyte sedimentation rate, cholesterol levels, triglyceride levels, glucose, insulin, and glycated hemoglobin (HbA1c) were evaluated from medical records. Waist circumference was defined as elevated > 94 cm in men and > 80 cm in women [21]; high blood pressure was defined as systolic blood pressure ≥ 130 mmHg and/or diastolic blood pressure ≥ 85 mmHg and/or use of antihypertensive drugs. Impaired glucose metabolism was defined as the existence of diabetes or impaired glucose tolerance (fasting glucose $\geq$ 100 mg/dL) and/or the use of anti-diabetic drugs. Hypertriglyceridemia was defined as triglyceride $\geq$ 150 mg/dL and/or use of drug treatment for elevated triglyceride levels. Low HDL cholesterol is defined as HDL cholesterol levels < 40 mg/dl in men and < 50 mg/dl in women and/or use of drug treatment for low HDL levels. Metabolic syndrome was diagnosed according to IDF 2009 criteria [21]. Elevated ferritin level was defined as higher than 200 ng/mL in women and higher than 300 ng/mL in men [22].

Bioelectrical impedance analysis (BIA) (Tanita BC420-MA (TANITA, Tokyo, Japan)) was used for determining the body composition of the participants.

Liver and spleen volumes were measured by magnetic resonance imaging (MRI).

The APRI score was calculated using the formula: APRI = [(AST level/ULN)/platelet count (10^9^/L)] × 100. The FIB-4 score was determined using the following formula: FIB-4: [age x AST/platelet count (10^9^/L) × √ALT] [23, 24]. Transferrin saturation (TS) of the patients were evaluated in terms of iron accumulation. Iron accumulation was excluded if TS was < 50%.

### Statistical analysis

Data were analyzed using IBM SPSS Statistics, version 21(SPSS, Inc.). Kolmogorov–Smirnov test was used to assess normality in addition to histograms and boxplots. Descriptive analyses were displayed using frequency tables for categorical variables and average and standard deviations for normal distributed variables. Non-normal distributions were presented as medians with interquartile ranges. The Mann–Whitney U test and student t-test were performed to compare groups (non-parametric and parametric data, respectively). Cross-group comparison for categorical variables was obtained using Chi-square/Fisher tests. Students paired T-test and Wilcoxon test were used to compare pre-ERT and post-ERT values (non-parametric and parametric data, respectively). In correlation analysis, the Spearman Test and Pearson test were used. A “*p*” value of < 0.05 was considered statistically significant.

## Results

### General features of the study group

Twenty-five Type 1 GD patients and twenty-five controls were recruited for the study. The main clinical features of the study population are shown in Table 1. Pre-ERT and post-ERT clinical and demographic features of the GD patients are also shown in Table 1***.*** The mean age of the patients was 41.5 $\pm$ 12.8 years (min–max: 21–75), with 56% female. The mean age of symptom onset was 24.8 ± 16.0 years (min–max: 2.5–70), while the mean age at diagnosis was 30.7 ± 15.1 years (min–max: 2.5–71). Fifteen patients (60%) had pathogenic c.1226A > G, p.Asn409Ser homozygous variant. Among them, seven patients also had c.1495G > A (p.Val499Met) homozygous pathogenic variant. Five patients had compound heterozygous c.1226A > G, p.Asn409Ser variants. The other 5 patients had a pathogenic variant other than p.Asn409Ser. All patients were receiving ERT. Twenty-three patients were under imiglucerase treatment and two were under taliglucerase treatment. The median duration of ERT was six (min–max: 0.5–17) years. Eight patients received enzyme replacement at a dose of 30 IU/kg, while 17 patients received 60 IU/kg (Table 1).

**Table 1:** Demographic, anthropometric, disease-related data and transient elastography measurements of the patients with Type 1 Gaucher disease and controls

	GD patients Pre-ERT (n = 25)	GD patients Post-ERT (n = 25)	Controls (n = 25)	*p*^¶^ value	*p*^#^ value
Demographic data					
Gender (F/M)	14/11	14/11	16/9	1.000	0.564
Age (years) mean±sd [min-max]	30.7 $\pm$ 15.1 [2.5–71]	41.5 $\pm$ 12.8 [21–75]	40.8 $\pm$ 11.4 [22–65]	** < 0.001**	0.843
Smoking n (%)	5 (20)	6 (24)	5 (20)	1.000	1.000
Presence of MetS n (%)	3 (12)	10 (40)	6 (24)	***0.016***	0.364
Anthropometric data					
BMI (kg/m^2^) mean±sd [min-max]	22.0 $\pm$ 2.7 [17.2–28.4]	23.8 $\pm$ 3.9 [17.2–31.8]	22.8 $\pm$ 4.9 [17.2–39.6]	** < *0.001***	0.425
Weight (kg) mean±sd [min-max]	57.3 $\pm$ 10.1 [34.3–82]	63.6 $\pm$ 11.9 [43.2–90.2]	62.4 $\pm$ 12.6 [44.8–89.2]	** < *0.001***	0.971
Gaucher disease related data					
Genotype n (%)			N/A		
c.1226A > G, p.Asn409Ser homozygous	15 (60)				
c.1226A > G, p.Asn409Ser heterozygous/ +	5 (20)				
c.1448 T > C (p.Leu483Pro)/c.1342G > C (p.Asp448His) compound heterozygous	2 (8)				
c.1214G > C (p.Ser405Thr) homozygous	2 (8)				
c.1604G > A (p.Arg535His) homozygous	1 (4)				
Age at the symptom onset (years) mean±sd [min-max]	24.8 $\pm$ 16.0 [2.5–70]				
Age at diagnosis (years) mean±sd [min-max]	30.7 $\pm$ 15.1 [2.5–71]				
Diagnosis lag time (months) median (IQR) [min-max]	21 (0–120) [0–298]				
Age at start of ERT (year) mean±sd [min-max]	N/A	35.5 $\pm$ 14.0 [15–71]			
Time to ERT from the beginning of the symptoms (months) median (IQR) [min-max]	N/A	108 (18–186) [1–432]			
Time to ERT from the diagnosis (months) median (IQR) [min-max]	N/A	12 (2.5–72) [1–432]			
Years on ERT median (IQR) [min-max]	N/A	6 (3–7) [0.5–17.0]			
Disease duration (years)	N/A	13 (7–24)			
ERT dose n (%) 30 IU/kg- 60 IU/kg	N/A	8 (32)–17 (68)			
Liver volume (cc) median (IQR) [min-max]	2222 (1667–3158) [1150–4500]	1607 (1401–1607) [1056–3150]	N/A	***0.001***	N/A
Spleen volume (cc) median (IQR) [min-max]	1404 (805.8–2700) [340–3920]	494 (351–838.3) [269–2310]	N/A	** < *0.001***	N/A
ESR (mm/h) median (IQR) [min-max]	20 (5.75–31.25) [2–103]	7 (4–14) [2–23]	7 (4–10) [3–27]	***0.001***	0.859
CRP (mg/L) median (IQR) [min-max]	1.7 (0.75–3.85) [0.3–30.9]	1.07 (0.5–2.95) [0.11–9.2]	1.5 (0.4–3.0) [0.1–15]	***0.063***	0.984
Ferritin (ng/mL) median (IQR) [min-max]	544 (256–907) [25–3733]	204 (48–581) [22–2645]	42.8 (26–95) [9.6–309]	** < *0.001***	** < 0.001**
Transferrin saturation (%) mean±sd	N/A	23 $\pm$ 10.1	26.2 $\pm$ 7.3	N/A	0.240
Liver fibrosis evaluation					
APRI score mean±sd	0.78 $\pm$ 0.55	0.28 $\pm$ 0.14	0.14 $\pm$ 0.08	** < 0.001**	**0.004**
FIB-4 score mean±sd	2.45 $\pm$ 2.1	1.16 $\pm$ 0.82	$0.79\pm$ 0.32	**0.002**	**0.047**
Transient Elastography (Fibroscan©) data					
Liver stiffness (kPa) median (IQR) [min-max]	N/A	6.6 (4.9–10.7) [3.3–21.5]	3.7 (3.3–4.4) [2.2–6.4]	N/A	** < 0.001**
Patients with liver stiffnes﻿s $\ge$ 7 kPa n (%)	N/A	11 (44)	0	N/A	** < 0.001**
Spleen stiffness (kPa) n = 21 median (IQR) [min-max]	N/A	17.6 (13.6–23,6) [7–60]	11.1 (9.1–18.8) [5–28.3]	N/A	**0.032**
CAP (dB/m) mean±sd [min-max]	N/A	234.7 $\pm$ 61.1 [115–400]	212.2 $\pm$ 43.3 [149–311]	N/A	0.138

n: number; sd: strandart deviation; IQR: interquartile range; min: minimum; max: maximum; GD: Gaucher disease; ERT: enzyme replacement therapy; F: female; M: male; BMI: body mass index; ESR: Erythrocyte sedimentation rate; CRP: C-reactive protein; APRI: AST to platelet ratio index; FIB-4: fibrosis-4; CAP: controlled attenuation parameter

^¶^Comparison between the pre-ERT and post-ERT parameters of patients with Gaucher disease *p* < 0.05 is statistically significant

^#^Comparison between the post-ERT parameters of patients with Gaucher disease and the controls. *p* < 0.05 is statistically significant

Weight, BMI, and MetS frequency increased after ERT (57.3 $\pm$ 10.1 vs 63.6 $\pm$ 11.9 kg, *p* < 0.001; 22.0 $\pm$ 2.7 vs. 23.8 $\pm$ 3.9 kg/m^2^, *p* < 0.001; 12% vs. 40%, *p* = 0.016, respectively). Despite the significant decrease in inflammation markers such as erythrocyte sedimentation rate and ferritin, the decrease in CRP was not significant [20 (5.75–31.25) vs 7 (4–14) mm/h, *p* = 0.001; 544 (256–907) vs. 204 (48–581) ng/mL, *p* < 0.001; 1.7 (0.75–3.85) vs. 1.07 (0.5–2.95) mg/L, *p* = 0.063, respectively]. Additionally, post-ERT serum ferritin levels were higher than the control group. Liver and spleen volumes of the GD patients were significantly decreased after the ERT. Transferrin saturation (TS) was below 50% in all patients except two patients receiving iron replacement therapy. These patients were not included in the evaluation. Mean TS was 23 $\pm$ 10.1% in GD patients and 26.2 $\pm$ 7.3% in controls (*p* = 0.240) (Table 1).

APRI and FIB-4 scores were within the normal range in most patients, but they were higher in the pre-ERT GD patients than post-ERT GD patients. Although post-ERT APRI and FIB4 scores declined, they were still higher than the controls (Table 1).

Elastography measurements of GD patients receiving ERT, and controls were performed cross-sectionally. Median liver and spleen stiffness were higher in GD patients than in controls [6.6 (4.9–10.7) vs. 3.7 (3.3–4.4) kPa; *p* < 0.001 and 17.6 (13.6–23.6) vs. 11.1 (9.1–18.8) kPa; *p* = 0.032). The mean CAP measurement was not different between the groups (Table 1).

### Factors associated with liver stiffness

GD patients under ERT were further divided into two subgroups: those with significant liver fibrosis (LS ≥ 7 kPa) (n = 11) and those without (n = 14) ***(***Table 2***).*** Liver fibrosis was more frequent in males (F/M: 3/8, *p* = 0.015). Weight, BMI, waist circumference, muscle mass, fat mass, and the presence of metabolic syndrome were not different between those with and without fibrosis. Serum AST, ALT, GGT, ferritin, and CRP levels, APRI score, and liver volume were found to be higher in patients with liver fibrosis (22.3 $\pm$ 5.3 vs 17.9 $\pm$ 4.7 U/L, *p* = 0.045; 23.4 $\pm$ 8.4 vs 15.8 $\pm$ 5.5 U/L, *p* = 0.019; 24.9 $\pm$ 11.5 vs 13.4 $\pm$ 6.6 U/L, *p* = 0.01; 578.8 (204–971) vs 89.2 (31.6–221.5) ng/mL, *p* = 0.03; 4.12 (2.82–5.08) vs 0.58 (0.34–1.28) mg/L, *p* = 0.011; 0.35 $\pm$ 0.18 vs 0.22 $\pm$ 0.07; *p* = 0.024; 1795 (1537–2100) vs 1520 (1302–1690) cc, *p* = 0.037). There was no difference in transferrin saturations between those with and without fibrosis (27.5 $\pm$ 6.8 vs. 20.5 $\pm$ 12.8%, *p* = 0.137).

**Table 2:** Comparison of patients with and without liver fibrosis

	Liver stiffness $\geq$ 7 kPa (n = 11)	Liver stiffness < 7 kPa (n = 14)	p* value
Age (years) mean±sd	45 $\pm$ 10	35 $\pm$ 14	0.193
Gender (%) F/M	3/8	11/3	***0.015***
Weight (kg) mean±sd	66.8 $\pm$ 12.7	61 $\pm$ 11.1	0.246
BMI (kg/m^2^) mean±sd	23.8 $\pm$ 3.6	23.8 $\pm$ 4.2	0.999
Presence of MetS %	54.5	28.6	0.187
Increased waist circumference %	63.7	57.1	0.742
Fat mass % mean±sd	22.6 $\pm$ 7.3	26.9 $\pm$ 8.3	0,187
Muscle mass % mean±sd	73.4 $\pm$ 6.8	68.8 $\pm$ 8.8	0.160
Fat free muscle mass % mean±sd	72.2 $\pm$ 18.4	73.3 $\pm$ 8.2	0.871
AST (U/L) mean±sd	22.3 $\pm$ 5.3	17.9 $\pm$ 4.7	***0.045***
ALT (U/L) mean±sd	23.4 $\pm$ 8.4	15.8 $\pm$ 5.5	***0.019***
GGT (U/L) mean±sd	24.9 $\pm$ 11.5	13.4 $\pm$ 6.6	***0.010***
Platelet count (10^3^/µL) median (IQR)	150 (118–244)	192(165–217)	0.273
Total-C (mg/dL) mean±sd	157.2 $\pm$ 38.4	166.7 $\pm$ 34.5	0.526
Triglyceride (mg/dL) median (IQR)	113 (86–129)	121(75–157)	0.722
LDL-C (mg/dL) median (IQR)	91(81–104)	96.5 (78.8–119)	1.000
HDL-C (mg/dL) mean±sd	35.6 $\pm$ 6.6	43.3 $\pm$ 13.5	0.077
HbA1c (%) mean±sd	5.3 $\pm$ 0.65	5.1 $\pm$ 0.57	0.451
Insulin (µIU/mL) mean±sd	8.5 $\pm$ 3.5	11.5 $\pm$ 2.4	0.226
Ferritin (ng/mL) median (IQR)	578.8 (204–971)	89.2 (31.6–221.5)	***0.030***
Transferrin saturation (%) mean±sd	27.5 $\pm$ 6.8	20.5 $\pm$ 12.8	0.137
CRP (mg/L) median (IQR)	4.12 (2.82–5.08)	0.58 (0.34–1.28)	***0.011***
APRI score mean±sd	0.35 $\pm$ 0.18	0.22 $\pm$ 0.07	***0.024***
FIB4 score mean±sd	1.47 $\pm$ 1.13	0.91 $\pm$ 0.32	0.091
Liver volume (cc) median (IQR)	1795 (1537–2100)	1520 (1302–1690)	***0.037***
Spleen volume (cc) median (IQR)	780 (367–1028)	416 (351–744)	0.177
CAP (dB/min) median (IQR)	239(183–291)	215(188–237)	0.396
Spleen stiffness (kPa) mean±sd	19.9 $\pm$ 19.9	20.1 $\pm$ 6.8	0.985
Splenectomy, (n)	3	0	0.23
Age at diagnosis (years) mean±sd	30.3 $\pm$ 16.4	31.1 $\pm$ 14.7	0.896
Disease duration (years) mean±sd	20.5 $\pm$ 14.4	13.8 $\pm$ 6.6	0.188
Age at the beginning of ERT (years) mean±sd	38.5 $\pm$ 13.2	33.1 $\pm$ 14.6	0.345
Years on ERT mean±sd	6.3 $\pm$ 4.3	5.2 $\pm$ 2.7	0.459
ERT dosage (n: 60/30 IU/kg)	10/1	7/7	***0.042***

sd: standart deviation; IQR: interquartile range; n: number; MetS: metabolic syndrome; KPa: kilopascal; F: female; M: male; BMI: body mass index; AST: aspartate transaminase; ALT: alanine transaminase; GGT: gamma-glutamyl transferase; HDL: high-density lipoprotein; -C: cholesterol; LDL: low-density lipoprotein; CRP: C-reactive protein; APRI: AST to platelet ratio index; FIB-4: fibrosis-4; CAP: controlled attenuation parameter; ERT: enzyme replacement therapy

^*^Statistically significant p values (< 0.05) are indicated in bold and italic in the table

The duration of ERT or genetic mutation type did not differ between those with and without fibrosis. Three patients who had undergone splenectomy previously had significant liver fibrosis (LS = 12.1; 9.3;14.6 kPa). Median liver stiffness was 12.1 kPa who underwent splenectomy and 6.15 kPa in those who did not (*p* = 0.058). When patients were classified according to enzyme dosage, most patients with significant liver fibrosis were receiving ERT at a dose of 60 IU/kg (*p* = 0.042) (Table 2).

**Fig. 1:**
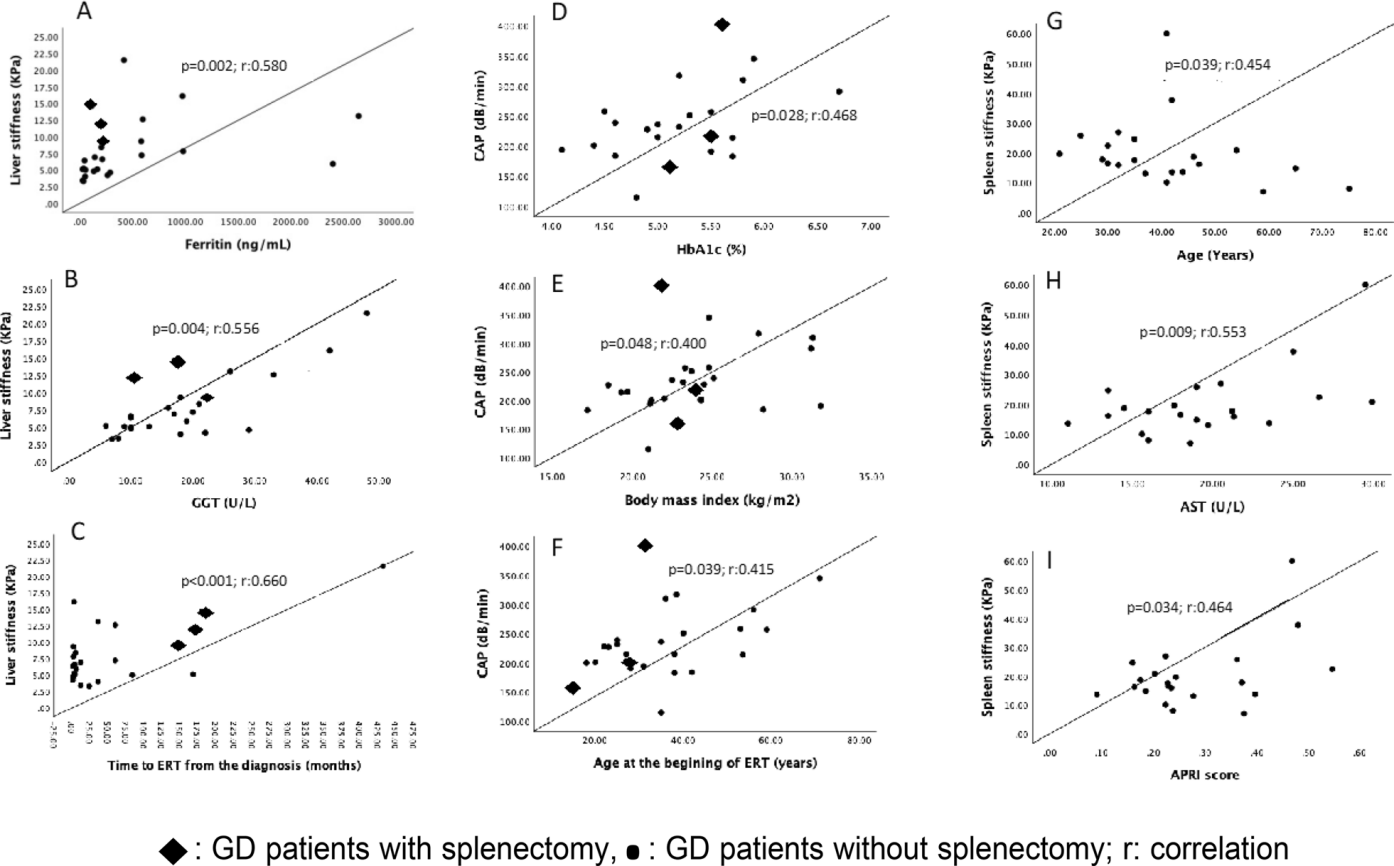
Scatter plots of variables with moderate or strong correlation with liver stiffness, CAP score and spleen stiffness (absolute value of Spearman correlation coefficient, r, IrI > 0.4). **A** Liver stiffness and ferritin (*p* = 0.002; r:0.580), **B** Liver stiffness and gamma-glutamyl transferase (GGT) (*p* = 0.004; r:0.556), **C** Liver stiffness and time to ERT from the diagnosis (*p* < 0.001; r:0.660), **D** CAP score and body mass index (*p* = 0.048; r:0.400), **E** CAP score and HbA1c (*p* = 0.028; r:0.468), **F** CAP score and age at the begining of ERT (*p* = 0.039; r: 0.415), **G** Spleen stiffness vs. age (*p* = 0.039; r:0.454), **H** Spleen stiffness vs. AST levels (*p* = 0.009; r: 0.553), **I** Spleen stiffness vs. APRI score (*p* = 0.034; r = 0.464

There was a positive correlation between liver stiffness and ALT, GGT, ferritin levels, FIB4 score, liver volume, time from diagnosis to ERT, and disease duration (Table 4). Scatter plots of variables with moderate or strong correlation with liver stiffness, CAP score and splenic stiffness shown in Fig. 1**.**

### Factors associated with CAP measurements

Gaucher patients under ERT were also grouped according to CAP measurements as those with significant steatosis (CAP measurement ≥ 250 dB/min; n = 8) and those without (n = 17) (Table 3). Patients with significant steatosis had higher BMI, fat mass percentage, hemoglobin A1c level, and liver volume (26.1 $\pm$ 3.58 vs 22.7 $\pm$ 3.56 kg/m^2^, *p* = 0.045; 29.2 $\pm$ 5.01 vs 23.1 $\pm$ 8.6%, *p* = 0.039; 5.6 $\pm$ 0.6 vs 4.99 $\pm$ 0.48%, *p* = 0.048; 1795 (1681–2315) vs 1548 (1308–1766) cc, *p* = 0.022, respectively). Muscle mass and fat-free muscle mass percentage were significantly lower in GD patients with steatosis (*p* = 0.041, *p* = 0.025, respectively). Metabolic syndrome was present in all cases with steatosis, and the frequency of metabolic syndrome was 17% in cases without steatosis (*p* > 0.001). Elevated waist circumferences ratio of patients with steatosis was also significantly higher than those without (87 vs 47%, *p* = 0.002). CAP measurements were higher in GD patients with MetS than those without (286 $\pm$ 59 vs 200 $\pm$ 31 dB/min, *p* < 0.001). In correlation analyses, CAP measurement was positively correlated with BMI, HbA1c level, and age at the beginning of the ERT (Table 4, Fig. 1).

**Table 3:** Comparison of patients with and without hepatic steat﻿osis

	CAP measurement $\ge$ 250 dB/min n = 8	CAP measurement < 250 dB/min n = 17	*p* value
Gender (n) F/M	4/4	10/7	0,679
Age (years) mean±sd	53 $\pm$ 12	36 $\pm$ 8	***0.006***
BMI (kg/m^2^) mean±sd	26.1 $\pm$ 3.58	22.7 $\pm$ 3.56	***0.045***
Weight (kg) mean±sd	69.8 $\pm$ 11.9	60.6 $\pm$ 11.1	***0.091***
Prevalance of MetS (%)	100	11.7	** < *0.001***
Increased waist circumference (%)	87	47	***0.002***
Fat mass (%) mean±sd	29.2 $\pm$ 5.01	23.1 $\pm$ 8.6	***0.039***
Muscle mass (%) mean±sd	66.2 $\pm$ 6.6	73.1 $\pm$ 8.2	***0.041***
Fat free muscle mass (%) mean±sd	64.5 $\pm$ 16.8	76.9 $\pm$ 8.6	***0.025***
ALT (U/L) mean±sd	20.55 $\pm$ 9.4	18.5 $\pm 7.1$	0.596
AST (U/L) mean±sd	17.9 $\pm$ 5.8	20.8 $\pm 4.95$	0.253
GGT (U/L) mean±sd	23.8 $\pm$ 14.3	16 $\pm$ 7.7	0.184
Platelet count(10^3^/µL) mean±sd	160 (118–244)	187 (166–218)	0.711
Total-C (mg/dL) mean±sd	180 $\pm$ 41.6	154 $\pm 3$ 0.7	0.148
Triglyceride (mg/dL)	146 (93–200)	108 (72–129)	0.086
LDL-C (mg/dL)	108.5 (86.5–138.5)	92 (79.5–105.5)	0.110
HDL-C (mg/dL) mean±sd	36.1 $\pm$ 8.6	41.7 $\pm$ 12.5	0.201
HbA1c (%) mean±sd	5.6 $\pm$ 0.6	4.99 $\pm$ 0.48	***0.048***
Ferritin (ng/mL) median (IQR)	496.9 (85.5–2042.8)	162.2 (45.2–271.5)	0.157
Transferrin saturation (%) mean±sd	22.2 $\pm$ 11.1	24.3 $\pm$ 11.2	0.707
CRP (mg/L) median (IQR)	2.84 (0,72–4.5)	0.88 (0.47–2.38)	0.166
APRI score mean±sd	0.31 $\pm$ 0.2	0.28 $\pm$ 0.12	0.683
FIB4 score mean±sd	1.59 $\pm$ 1.32	0.96 $\pm$ 0.33	0.219
Liver volume(cc) median (IQR)	1795 (1681–2315)	1548 (1308–1766)	***0.022***
Liver stiffness (kPa) median (IQR)	10.7 (4.48–15.4)	6.4 (4.9–8.1)	0.215
Spleen stiffness (kPa) median (IQR)	14.2 (7.8–27.2)	18.7 (15.9–24.7)	0.112
Splenectomy (n)	1	2	0.958
Age at diagnosis (years) mean±sd	41 $\pm$ 19	26 $\pm$ 10	0.069
Disease duration (years) mean±sd	19.1 $\pm$ 13.7	15.6 $\pm$ 10.9	0.535
Age at the beginning of ERT (years) mean±sd	49 $\pm$ 14	30 $\pm$ 10	***0.006***
Years on ERT mean±sd	4.6 $\pm$ 2.6	6.2 $\pm$ 3.6	0.273
ERT dosage (n: 60/30 IU/kg)	6/2	11/6	0.487

sd: standart deviation; IQR: interquartile range; n: number;F: female; M: male; BMI: body mass index; AST: aspartate transaminase; ALT: alanine transaminase; GGT: gamma-glutamyl transferase; HDL: high-density lipoprotein; -C: cholesterol; LDL: low-density lipoprotein; CRP: C-reactive protein; APRI: AST to platelet ratio index; FIB-4: fibrosis-4; CAP: controlled attenuation parameter; ERT: enzyme replacement therapy

^*^Statistically significant p values (< 0.05) are indicated in bold and italic in the table

**Table 4:** Correlation analyses of liver, spleen stiffness and CAP measurements

	Liver stiffness	CAP measurement	Spleen stiffness
Age (years)	p = 0.143 (r:0.301)	p = 0.076 (r:0.362)	***p* = *0.039 (r:-0.454)***
BMI (kg/m^2^)	p = 0.867 (r:0.035)	***p* = *0.048 (r:0.400)***	p = 0.743 (r:-0.454)
Weight (kg)	p = 0.289 (r:0.221)	p = 0.398 (r:0.177)	p = 0.161 (r:0.317)
Waist circumference (cm)	p = 0.130 (r:0.311)	p = 0.145 (r:0.300)	p = 0.684 (r:0.094)
Hip circumference (cm)	p = 0.620 (r:0.104)	p = 0.446 (r:0.160)	p = 0.472 (r:-0.166)
Fat mass (%)	p = 0.673 (r:-0.091)	p = 0.140 (r:0.311)	p = 0.982 (r:0.005)
Muscle mass (%)	p = 0.648 (r:0.098)	p = 0.073(r:-0.373)	p = 0.810 (r:0.056)
Fat free muscle mass (%)	p = 0.638 (r:-0.101)	p = 0.147 (r:-0.305)	p = 0.980 (r:-0.006)
AST (U/L)	p = 0.419 (r:0.169)	p = 0.603 (r:-0.109)	***p* = *0.009 (r:0.553)***
ALT (U/L)	***p* = *0.021 (r:0.460)***	p = 0.920 (r:0.021)	***p* = *0.027 (r:0.483)***
GGT (U/L)	***p* = *0.004 (r:0.556)***	p = 0.462 (r:0.152)	p = 0.904 (r:-0.024)
Platelet count (10^3^/µL)	p = 0.733 (r:-0.072)	p = 0.384 (r:-0.182)	p = 0.557 (r:-0.136
Total-C (mg/dL)	p = 0.793 (r:-0.055)	***p* = *0.045 (r:-0.404)***	p = 0.221 (r:-0.279)
Triglyceride (mg/dL)	p = 0.162 (r:-0.288)	p = 0.114 (r:0.324)	p = 0.343 (r:-0.218)
LDL-C (mg/dL)	p = 0.839 (r:-0.043)	p = 0.339 (r:0.199)	p = 0.211 (r:-0.285)
HDL-C (mg/dL)	p = 0.223 (r:-0.253)	p = 0.198 (r:-0.266)	p = 0.729 (r:-0.081)
HbA1c (%)	p = 0.664 (r:0.098)	***p* = *0.028 (r:0.468)***	p = 0.894 (r:-0.033)
Ferritin (ng/mL)	***p* = *0.002 (r:0.580)***	p = 0.462 (r:0.152)	p = 0.942 (r:0.017)
Transferrin saturation (%)	p = 0.126 (r:0.354)	p = 0.920 (r:0.024)	p = 0.801 (r:-0.066)
CRP (mg/L)	p = 0.067 (r:0.380)	p = 0.070 (r:0.376)	p = 0.505 (r:-0.154)
APRI score	p = 0.126 (r:0.314)	p = 0.510 (r:0.138)	***p* = *0.034 (r:0.464)***
FIB4 score	***p* = *0.034 (r:0.423)***	p = 0.271 (r:0.229)	p = 0.662 (r:-0.101)
Liver volume (cc)	***p* = *0.024 (r:0.469)***	p = 0.102 (r:0.349)	p = 0.723 (r:0.087)
Spleen volume (cc)	p = 0.101(r:0.377)	p = 0.230 (r:0.281)	p = 0.158 (r:0.337)
Liver stiffness (kPa)		p = 0.402 (r:0.175)	p = 0.845 (r:0.045)
CAP (dB/min)	p = 0.400 (r:0.175)		p = 0.996 (r:-0.001)
Spleen stiffness (kPa)	p = 0.845(r:0.045)	p = 0.996 (r:-0.001)	
Time from diagnosis to ERT (months)	***p* < *0.001 (r:0.660)***	p = 0.771 (r:0.061)	p = 0.978 (r:0.006)
Age at the beginning of ERT (years)	p = 0.059 (r:0.383)	***p* = *0.039 (r:0.415)***	p = 0.313 (r:-0.231)
Disease duration (years)	***p* = *0.018 (r:0.469)***	p = 0.888 (r:0.030)	p = 0.092 (r:-0.377)
Years on ERT	p = 0.685 (r:0.85)	p = 0.374 (r:-0.186)	p = 0.558 (r:-0.136)

r: correlation coefficient; BMI: body mass index; AST: aspartate transaminase; ALT: alanine transaminase; GGT: gamma-glutamyl transferase; HDL: high-density lipoprotein; -C: cholesterol; LDL: low-density lipoprotein; CRP: C-reactive protein; APRI: AST to platelet ratio index; FIB-4: fibrosis-4; CAP: controlled attenuation parameter; ERT: enzyme replacement therapy

^*^Statistically significant p values (< 0.05) are indicated in bold and italic in the table

### Factors associated with spleen stiffness

A clear cut-off value has not been currently defined in the literature for spleen stiffness. Splenic stiffness could not be measured in three patients due to a history of splenectomy and in one patient with a splenic artery stent placed for aneurysm management. Among the remaining patients receiving ERT, the median splenic stiffness was 17.6 kPa. Using this median value as a threshold value, patients with splenic stiffness ≥ 17.6 kPa were significantly younger (35.5 ± 9.6 vs 47.2 ± 14.7 years, *p* = 0.048) and exhibited slightly higher levels of ALT, although within the normal range (22.5 ± 7.4 U/L vs 15.5 ± 7.5 U/L, *p* = 0.046). There was no statistically significant difference in terms of other demographic, metabolic, or disease-specific parameters. Splenic stiffness was negatively correlated with age and positively correlated with AST, ALT levels, and APRI scores (Table 4, Fig. 1).

## Discussion

Transient elastography (TE) is a widely used method for chronic liver disease and hepatic steatosis. Despite its widespread use, there are few studies evaluating steatotic liver disease and liver/spleen stiffness with TE in GD [10–13, 20]. In this retrospective, cross-sectional study of adult type 1 GD patients undergoing ERT, TE revealed significantly elevated liver and spleen stiffness compared to matched controls. ERT over a median duration of six years was associated with improvements in liver and spleen volumes, APRI and FIB-4 scores, inflammatory markers as well as increased BMI and the prevalence of metabolic syndrome. Despite these improvements significant liver fibrosis persisted in 44% of patients.

Previous studies evaluating liver fibrosis in GD using TE have reported variable rates of fibrosis across different cohorts from Italy, Poland, the Netherlands, and Israel [10, 11, 13, 20]. Bohte et al. [20] reported liver stiffness values of 4.9 kPa in healthy controls, 8.8 kPa in GD individuals with splenectomy, and 4.8 kPa in GD patients without splenectomy, identifying splenectomy as a risk factor for subsequent liver fibrosis. In addition, Nascimbeni et al. [11] reported that the median liver stiffness was 4.6 kPa in type 1 GD patients. Significant fibrosis was detected in 19% of cases and 57% of these patients had splenectomy [11]. In contrast to these findings, Webb et al. [10] reported that 13 out of 42 GD patients had a history of splenectomy, nevertheless splenectomy was not identified as a risk factor for liver fibrosis. In their cohort, liver stiffness was 7.1 kPa in GD patients and 5 kPa in controls [10]. In our study, consistent with the aforementioned studies, median liver stiffness was higher in GD patients than in matched controls (6.6 vs 3.7 kPa; *p* < 0.001). However, unlike prior studies, there were only three patients who underwent splenectomy [10, 11, 20]. Significant liver fibrosis was present in all of these three patients. Due to the low number of splenectomized individuals in our cohort, we were unable to statistically evaluate splenectomy as a risk factor for increased liver stiffness.

Significant liver fibrosis has been defined as a liver stiffness measurement of ≥ 7 kPa in both previous studies and the present study. The prevalence of significant liver fibrosis among type 1 GD patients has been reported to range from 19 to 50% [10, 11, 13, 20]. In the present study, significant liver fibrosis was found in 44% of the patients. In both the studies by Lipinski [13] and Nascimbeni et al. [11] a high proportion of patients demonstrated c.1226A > G, p.Asn409Ser pathogenic variant (100% and 89%, respectively). On the other hand, although 80% (n = 20) of the patients in our cohort had c.1226A > G, p.Asn409Ser pathogenic variant, interestingly, 7 of these patients had both c.1226A > G, p.Asn409Ser and c.1495G > A (p.Val499Met) homozygous pathogenic variants. These genotypic differences may be the reason for the higher incidence of significant liver fibrosis compared to other studies. In addition, in the study of Nascimbeni et al. [20] four patients had active alcohol consumption that could lead to chronic liver disease, which may have affected liver stiffness measurements. One patient in our study had chronic hepatitis B infection. However, no significant liver fibrosis was detected in this patient (LS = 5.9 kPa). In the study by Bohte et al. [20] the c.1226A > G, p.Asn409Ser pathogenic variant was present in all patients, but the proportion of splenectomized patients in the cohort was quite high at 50% and concomitant hemochromatosis was also reported in the study group. This may explain the higher frequency of liver fibrosis in the cohort of Bohte et al. [20] compared to other studies.

In a recent 2025 study, Starosta et al. [16] used transient elastography (TE) to assess liver stiffness in Gaucher disease and found significant correlations with AST and GGT levels, supporting our findings. They also noted that APRI and FIB4 scores have limited diagnostic value in this population. Similarly, our study showed positive correlations between ALT, GGT, and liver stiffness. While APRI was not associated, FIB4 had a moderate positive correlation. These scores were generally higher in patients with fibrosis but often remained within normal limits, indicating limited utility. Starosta et al. [16] also developed the Gaucher Liver Fibrosis Score (GLFS), highlighting the need for disease-specific risk stratification tools.

Serum ALT, GGT, ferritin levels, liver volume, disease duration, and the time from diagnosis to ERT were also positively correlated with liver stiffness in correlation analyses. The positive correlation between ferritin and liver stiffness, along with higher CRP levels in the fibrosis group -even within the normal range- suggests that ongoing low-grade inflammation may contribute to the development of liver fibrosis. Although it is pathophysiologically possible, it is important to note that causality cannot be definitely established from our data. Whether inflammation contributes to fibrosis or results from it remains unclear. The median time from diagnosis to ERT was 12 months and was positively correlated with liver stiffness. A patient who did not attend follow-up visits after diagnosis and remained untreated for 432 months had the highest liver stiffness (21.5 kPa), highlighting the importance of timely intervention. The patient was evaluated for liver cirrhosis. There were no clinical or laboratory findings of cirrhosis or portal hypertension. No esophageal varices were found in endoscopic examination. Other causes of liver fibrosis were ruled out, and liver involvement was attributed to Gaucher disease; since there were no signs of cirrhosis, and the patient is being closely monitored. Ferritin may be increased as an acute-phase reactant in inflammation but may also be increased in iron accumulation. In our study, transferrin saturation was also evaluated, except for 2 Gaucher patients and 2 controls receiving iron replacement therapy and was found to be below 50%. Therefore, ferritin elevation due to iron accumulation was excluded [25].

The disease severity scores of patients with liver fibrosis were higher in the study by Nascimbeni et al. [11]. In our study, 10 of 11 patients with liver fibrosis were receiving ERT at a dose of 60 IU/kg. The dose of ERT indirectly indicates disease severity. In this respect, our findings are consistent with previous studies.

In two previous reports, CAP measurements in GD patients were associated with BMI, weight, waist circumference, hypertension, and metabolic syndrome [12, 13]. These studies reported significant hepatic steatosis in 22–40% of GD patients. Consistent with these findings, we observed significant hepatic steatosis in 32% of our GD patient cohort, all of whom had metabolic syndrome. Compared to patients without steatosis, those with steatosis had higher waist circumference, fat mass, HbA1c levels, and liver volumes, but lower muscle mass and fat-free muscle mass. None of the patients consumed alcohol. Aside from liver volume, CAP measurements showed no relationship with other Gaucher disease-related parameters. Although no significant differences were observed in CAP measurements, there was a significant increase in the prevalence of MetS after ERT, rising from 12 to 40%. CAP scores were higher among GD patients who met the criteria for metabolic syndrome, suggesting a possible link between metabolic alterations and hepatic steatosis.

There are several theories regarding the development of hepatic steatosis in GD patients. It is known that disorders in the pathways of sphingolipid metabolism, such as GD, lead to insulin resistance. Ceramide and many sphingolipids form the basis of the membrane lipid structure. This lipid structure is required for insulin signaling. Ganglioside GM3 accumulated in GD negatively affects the insulin receptor in the membrane lipid structure, reduces insulin sensitivity, and causes insulin resistance [26, 27]. Apart from this mechanism, untreated GD is a highly catabolic process. Systemic inflammation may lead to an increased resting energy expenditure. If adequate caloric intake is not maintained to meet this energy expenditure, which increases by approximately 44%, growth retardation in children and loss of muscle mass in adults may occur [9, 28]. Basal metabolic rate decreases markedly after ERT. ERT has been associated with insulin resistance and weight gain [29, 30]. In our study, the weight and BMI of the patients increased after ERT, and the frequency of metabolic syndrome increased from 12 to 40%. The presence of metabolic syndrome is a risk factor for the development of hepatic steatosis in Gaucher patients as in the general population. It may be recommended to examine for hepatic steatosis and metabolic syndrome, especially in patients with elevated waist circumferences.

Transient elastography measurement of splenic stiffness has been used in conditions such as chronic liver disease and myelofibrosis. It is known to be associated with portal hypertension. Webb et al. [10] previously evaluated splenic stiffness with 2-dimension shear wave elastography (SWE) and TE. SWE and TE measurements reported in kilopascals (kPa), may report different values even within the same individual. Therefore, it is essential to clearly specify the elastography technique used when interpreting and comparing stiffness data [31]. In our study, the median splenic stiffness values were lower than those measured with TE by Webb et al. [10]. Specifically, the median splenic stiffness in GD patients was 17.6 kPa in our cohort versus 35 kPa in the study by Webb et al., while the control group values were 11.1 kPa versus 16.95 kPa, respectively. This discrepancy may be explained by the demographic characteristics of the study populations. In our study, participants were generally younger, and all patients were receiving ERT which may contribute to reduced spleen stiffness. In our cohort, splenic stiffness was positively correlated with alanine aminotransferase (ALT) levels and negatively correlated with age. However, no significant correlation was observed between splenic stiffness and GD related clinical or genetic parameters.

One limitation of our study is that liver and spleen stiffness, as well as CAP measurements, could not be performed prior to the initiation ERT, as TE was not in use or widely available at that time. Evaluating treatment effectiveness through TE would be more robust if pre- and post-ERT measurements were available, particularly in treatment-naïve patients.

Another limitation is the relatively small sample size. However, considering that GD is a rare disorder, the number of included patients is reasonable and comparable to previous studies in literature. Nevertheless, the limited sample size precluded post-hoc subgroup analyses and may limit the statistical power, especially for variables with borderline significance. Therefore, findings with marginal *p*-values should be interpreted with caution. Although the study has a retrospective design, GD requires close clinical follow-up and nearly all relevant clinical and laboratory parameters are routinely assessed every six months in our center. National reimbursement regulations mandate biannual assessment of most clinical and laboratory parameters, resulting in minimal missing data.

## Conclusions

In conclusion, TE is a valuable noninvasive method for assessing liver fibrosis and hepatic steatosis in patients with type 1 GD and may serve as a useful adjunct to routine monitoring in this population. The detection of fibrosis is particularly relevant in individuals with a prolonged disease course and elevated inflammatory markers. TE is also useful for detecting hepatic steatosis, particularly in overweight GD patients with metabolic syndrome. Future larger scale studies incorporating both pre- and post-ERT TE measurements, along with broader demographic diversity, are warranted to better elucidate the long-term effects of ERT on liver and spleen stiffness.

## Data Availability

Patient data is available from Ummu Mutlu upon reasonable request.

## Abbreviations

GD: Gaucher disease
ERT: Enzyme replacement therapy
TE: Transient elastography
CAP: Controlled Attenuation Parameter
MetS: Metabolic syndrome
ALT: Alanine transaminase
APRI: AST to platelet ratio index
FIB-4: Fibrosis-4
GGT: Gamma-glutamyl transferase
*GBA1*: Glucocerebrosidase 1
MAFLD: Metabolic-associated fatty liver disease
BMI: Body-mass index
M: Medium
XL: X-large
LS: Liver stiffness
SS: Spleen stiffness
KPa: Kilopascal
HbA1c: Glycated hemoglobin
HDL: High-density lipoprotein
LDL: Low-density lipoprotein
TS: Transferrin saturation
AST: Aspartate transaminase
CRP: C-reactive protein
-C: Cholesterol
